# Endocrine Alterations Are the Main Determinants of Cardiac Remodelling in Restrictive Anorexia Nervosa

**DOI:** 10.5402/2011/171460

**Published:** 2011-07-02

**Authors:** Guido Carlomagno, Valentina Mercurio, Antonio Ruvolo, Ignazio Senatore, Irina Halinskaya, Valeria Fazio, Flora Affuso, Serafino Fazio

**Affiliations:** ^1^Department of Internal Medicine, Cardiovascular and Immunological Sciences, University of Naples Federico II, Via Pansini 5, 80131 Naples, Italy; ^2^Department of Psychiatry, University of Naples Federico II, Via Pansini 5, 80131 Naples, Italy

## Abstract

*Objective*. Anorexia nervosa is a condition of reduced hemodynamic load, characterized by varying degrees of cardiac remodelling, only in part related to reduced body mass; the mechanism for such variability, as well as its clinical significance, remains unknown. Aim of the study was to assess the possible influence of a great number of clinical, biochemical, and endocrine factors on cardiovascular parameters in restrictive anorexia nervosa. *Method*. Twenty-five female patients hospitalized for restrictive anorexia nervosa underwent extensive cardiovascular, clinical, and biochemical evaluation. *Results*. Height-adjusted and cardiac workload-matched left ventricular mass was significantly related to several endocrine parameters, blood pressure, and vasoreactivity. On multivariate analysis, IGF/GH ratio and systolic blood pressure were the only independent predictors of height-adjusted ventricular mass (adj-*R* ^2^ = 0.585; *P* = 0.001); when matching for cardiac workload, left ventricular mass was independently predicted only by GH and FT3 levels. All effects were independent of patient's weight and BMI. *Conclusions*. Indices of endocrine impairment seem to be the most relevant determinants of left ventricular hypotrophy in anorectic patients, apparently independent of reduced hemodynamic load and BMI. In particular, IGF/GH ratio and FT3 seem to particularly affect left ventricular mass in this population.

## 1. Determinants of Cardiac Remodelling in Restrictive Anorexia Nervosa

Anorexia nervosa (AN) is a common psychiatric disorder, characterized by weight loss, protein-energy malnutrition, and severe organic complications leading to increased long-term morbidity and mortality; despite the prevalence and severity of this condition, clinicians still rely mainly on BMI and clinical assessment in the risk stratification of AN patients [1]. Most of the organic abnormalities described in anorectic patients are traditionally regarded to as adaptive mechanisms of protection against chronic starvation [2]. Several studies have explored the cardiac findings in the setting of AN: frequent morphological features include mitral valve prolapse, mild pericardial effusion, and a reduced left ventricular mass [3–8]. Although the latter could be interpreted as an adaptive response to the reduced hemodynamic needs during AN, researchers agree that ventricular remodelling appears to be extremely variable among patients, apparently independent from the extent of weight loss and other traditional markers of malnourishment.

To date, no study has tried to specifically identify the determinants of such variability in left ventricular mass and cardiovascular pattern of AN patients.

## 2. Methods

### 2.1. Subjects

Twenty-five women with restrictive-type AN were enrolled during hospitalization at the local psychiatric institution. Diagnosis of AN was confirmed by two independent consultant psychiatrists following current DSM-IV criteria [9]. Binging and/or purging behaviours were specifically excluded in all patients. Patients with previously diagnosed cardiac and/or endocrine disease were excluded. All patients reported a sedentary lifestyle and no cardiac symptoms at rest or upon effort.

Most patients underwent both cardiovascular assessment and blood sampling before the beginning of any therapeutic intervention; in the minority of patients enrolled during urgent hospitalization, blood sampling occurred before treatment, while cardiovascular assessment was delayed to two to three days after start of refeeding. The study conformed to the Declaration of Helsinki. Patients and their families gave informed consent to the procedures.

### 2.2. Measurements

Patient's weight and body composition were studied by means of a footpad bioelectrical impedance analyzer (Tanita TBF-215 device, Tanita Corp., Japan) by using manufacturer-provided formulas. 

Blood pressure was measured after ten minutes of rest in a sitting position, using a standard adult or pediatric arm-cuff, according to the subject's arm circumference.

A complete echocardiographic examination was performed using a commercially available system (Aplio CV, Toshiba Corporation, Otawara, Japan). Left ventricular mass (LVM) was estimated using M-Mode measurements of left ventricular wall thickness and diameters, following the recommendations of the American Society of Echocardiography [10]; the estimate of myocardial mass was then normalized by body surface area (LVMi) and height in meters to the power of 2.7 (LVMh) [6]. 

In order to take into account the reduced hemodynamic load of this particular population, weight-independent, height- and stroke-work-predicted left ventricular mass was calculated for each subject by means of a validated formula, as previously described by others in a similar population [7]; LVM% was then defined as follows: (observed LVM/predicted LVM)∗100. LVM% was accordingly defined as “inadequate” using reference values from the same study (observed LVM less than 73% of predicted LVM).

Endothelial-dependent dilation was assessed with the flow-mediated dilation test (FMD) of the brachial artery with Doppler echocardiography, according to the recommendations of the International Brachial Artery Reactivity Task Force [11]. The upper arm was occluded for 5 min resulting in a reactive hyperemia after the release of the cuff, and the increased shear stress led to endothelial-mediated vasodilatation. FMD was measured with the same ultrasound scanner with a 7.5 MHz linear transducer (Toshiba PLT-704AT), soon after the echocardiographic exam.

Cardiac evaluation also included 24-hour ambulatory ECG monitoring, with subsequent offline analysis of heart rate variability (HRV) both in frequency and time domain, as described elsewhere [12].

For each instrumental technique, the same investigator performed and analyzed all assessments.

Venus blood samples were collected in the morning, after an overnight fast (14 hrs), for measurement of insulin-like growth factor 1 (IGF-1), growth hormone (GH), cortisol, ACTH, thyrotropin (TSH), FT_3_, FT_4_, prolactin, and DHEAS. Serum GH was assayed with an immunoradiometric assay IRMA method and serum IGF-I with an RIA using a monoclonal antibody after acid-ethanol extraction. Evaluation of plasma TSH levels was performed by an ultrasensitive immunoradiometric assay with a detection limit of 0.05 mU/L. Serums FT_4_ and FT_3_ were measured using the Lisophase Kits (Bouty). All samples were also analyzed for common biochemistry, electrolytes, and complete blood count.

### 2.3. Statistical Analysis

Data are expressed as means ± SDs. Analysis was performed using SPSS version 12 (SPSS Inc., Chicago, IL). Log-transformed values were used for statistical analysis of the ratio of IGF-1 to GH (log IGF1/GH), as already described by others [13]. Two-sided unpaired *t*-test was used for difference between subgroups. The Pearson (r) correlation method was used for correlation analysis between continuous variables. A *P* < 0.05 was considered significant. Linear regression analysis was performed to identify cofactors predictive of lower LVMh and LVM%; significantly associated dependent variables were then analyzed in a stepwise multiple regression model.

## 3. Results

Patient characteristics, echocardiographic, electrocardiographic, vasoreactivity, biochemical, and endocrine parameters are shown in Table 1. Our population exhibited very low absolute values of LVM, although only 7 out of 25 patients (28%) showed “inadequately” low LVM% when matched for height and cardiac workload, using the same cut-offs as in Romano et al. [7]. 

Subgroup analysis comparing patients with “inadequately” low LVM% (see Section 2) only showed significant differences in terms of GH, logIGF/GH, and FT3 levels (see Table 2). No difference was found regarding BMI and other anthropometric and biochemical variables

**Figure 1:**
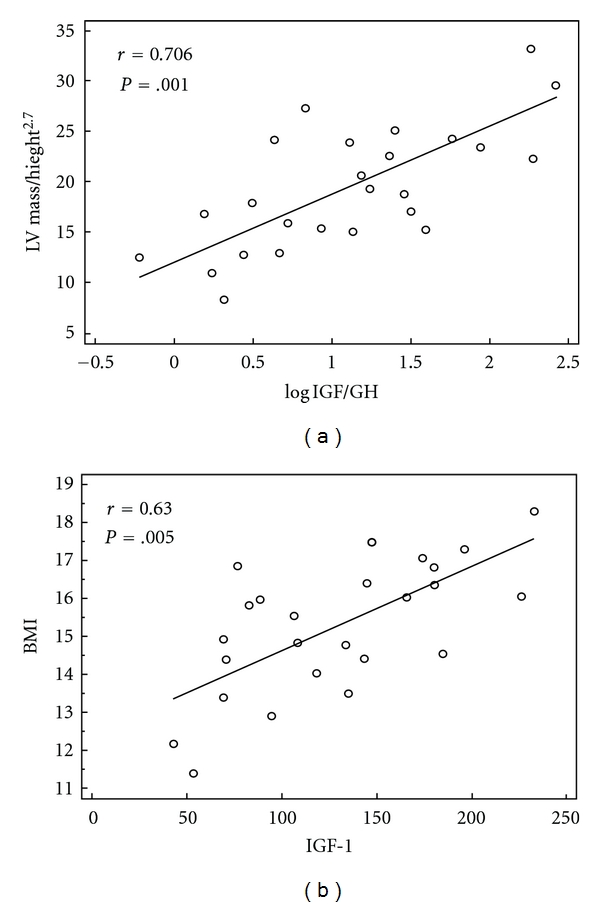
Scatter plots of LVMh versus logIGF-1/GH (a) and BMI versus serum IGF-1 (b).

Correlation analysis revealed strong, significant correlations between logIGF-1/GH and both LVMi, LVMh, and LVM% (resp.,  *r* = 0.638, *r* = 0.706, and *r* = 0.618; *P* = 0.001 for LVMh; Figure 1(a)). Other significant correlations were found for LVMh with SBP, FMD values, and GH levels (resp., *r* = 0.603; *r* = 0.489; *r* = −0,54; *P* < 0.05 for all). 

Among hormonal parameters, only IGF-1 (*r* = 0.630; *P* = 0.005; Figure 1(b)) and cortisol (*r* = −0.579; *P* = 0.01) were significantly correlated with the patient's BMI, while no significance was found for GH, logIGF1/GH, DHEAS, and thyroid hormones.

Linear regression analysis was performed looking for predictors of LVMh among all explored variables. Covariates found significant at univariate analysis were SBP, FMD, GH, logIGF-1/GH (*P* < 0.05 for all). These were used for multiple, backward, stepwise linear regression; in such analysis logIGF-1/GH, and SBP revealed as the only independent predictors of LVMh and LVMi (adj-*R* ^2^ = 0.585; *P* = 0.001). 

A further analysis was performed using LVM% as the dependent variable; significant univariate cofactors were BMI, GH levels, logIGF-1/GH and FT_3_. On backward stepwise multiple regression analysis, reduced FT_3_, and elevated GH levels remained the only independent predictors of low LVM% (adj-*R* ^2^ = 0.384; *P* < 0.02). 

No significant correlation was found between HRV parameters, body composition and any of the hormones measured.

## 4. Discussion

This is the first study to systematically address the determinants of cardiac alterations in AN. The patterns of cardiac and endocrine alterations in our patients substantially resemble those reported in previous studies. Although IGF-1 and cortisol correlated well with body weight and BMI, not all of the variability in IGF1 : GH ratio and thyroid hormone levels seems attributable to body composition alone; this phenomenon has recently been described in a larger report, and it might account for some of the clinical and prognostic discrepancy among patients with similar BMIs [14]: individuals with similar BMIs may show very different degrees of organic compromise, as indicated by heterogeneous endocrine and cardiac phenotypes, and such differences might affect the patient's prognosis.

The present results suggest a strong association between high GH levels, low IGF-1, low FT_3_, and reduced LVM in anorectic patients. Interestingly, this association seems to act independent of body weight, BMI, left ventricular afterload and peripheral markers of malnutrition. It is therefore likely that the very low values of LVM observed in some emaciated patients do not exclusively reflect reduced hemodynamic requirements due to low body mass, as elsewhere postulated, but also mirror a more complex alteration of the metabolic and endocrine status of the whole organism. 

Actually, only a subgroup of patients in our cohort showed “inadequately” low LVM (about 30% of the subjects); this is in line with the most important studies in the field to date, and our findings propose an intriguing explanation of this heterogeneity [7, 8]. These patients showed a worse overall status, with a trend towards lower BMI and longer history of disease; though, the only significant differences between the two groups were in terms of endocrine markers. It is well known that GH, FT_3_ and IGF-1 exert potent physiologic trophic effects on the myocardium, both in health and disease; it is therefore reasonable that only those patients who develop a multiple endocrine dysfunction with peripheral resistance to GH and low FT_3_ levels besides weight loss also show inadequately low cardiac mass. Although it is common sense to conceive the endocrine changes of anorexia as adaptive responses to starvation, such changes have recently been demonstrated to predict high short-term probability of severe organic complications, in a fashion independent of BMI and other “traditional” markers [14]; our study confirms that endocrine and cardiac abnormalities in these populations are, to a certain extent, unrelated to body weight and BMI. Hence, a low cardiac mass could represent a useful supplemental clinical marker of systemic malnourishment. Of note, one previous study described concordant reversal of both cardiac hypotrophy and low IGF-1 levels after weight gain in a few adolescent AN patients [15].

Our findings also put forward again the question whether the alterations in the somatotropic axis are among the main actors of the “metabolic blockade” typical of the most compromised AN patients, in whom muscle catabolism can continue despite aggressive nutritional support. Many groups have hypothesized a beneficial effect of GH and IGF-1 administration on appetite, muscle metabolism, and bone mass density in AN [16]. The studies brought equivocal results; at least for IGF-1 therapy, this was also due to short treatment duration and/or low doses employed, aimed mainly at observing an improvement in bone density or surrogates of bone turnover [17, 18]. Further studies with higher doses are being designed by us and others.

## 5. Study Limitations

The present study is mainly limited by the small number of subjects examined; however, both cardiac and endocrine findings in our patients were reasonably compatible with those of previous works. The absence of a control group was intentional, as all of the explored parameters had already been individually studied in similar populations. Growth hormone status is best assessed by means of multiple measurements throughout the day and overnight; this was not performed due to logistical reasons in this particular population. Of course, no causative relationship can be asserted between GH/IGF1 imbalance, FT_3_ levels and cardiac hypotrophy; similarly, no prognostic effect can be asserted from our data alone. Larger, longitudinal follow-up studies in similar populations will be of help in this regard.

## 6. Conclusions

Our results confirm that left ventricular remodelling is very variable in AN and show that parameters of GH-resistance and low FT_3_ levels seem to be the best predictors of low left ventricular mass beyond hemodynamic load and BMI. Therefore, cardiac phenotype seems a valuable candidate parameter for prognostic stratification in this patient population.

## Figures and Tables

**Table 1:** Patient characteristics, echocardiographic, endocrine, and electrocardiographic parameters (*n* = 25).

Age (years)	24 ± 9	Tryglycerides (mg/dL)	70 ± 18
SBP (mm Hg)	92 ± 8	Hemoglobin (g/dL)	11,9 ± 1,5
DBP(mm Hg)	72 ± 6	GH (ng/mL)	11.7 ± 16.6
HR (bpm)	58 ± 10	IGF-1 (ng/mL)	127 ± 53
Weight (kg)	39 ± 5	logIGF-1/GH	1.26 ± 0.60
BMI	15.2 ± 2.0	Cortisol (ng/mL)	168 ± 36
Fat mass (kg)	5.2 ± 2.0	TSH (*μ*U/mL)	2.0 ± 0.8
Lean mass (kg)	32.7 ± 4.0	FT3 (pg/mL)	1.8 ± 0.5
RWT	0.35 ± 0.06	FT4 (ng/dL)	0.9 ± 0.1
LVM (g)	75 ± 19	Prolactin (ng/mL)	9.9 ± 7.4
LVMi (g/m^2^)	57 ± 14	DHEAS (*μ*g/dL)	105 ± 46
LVMh (g/m^2.7^)	22 ± 6	FMD (%)	13.9 ± 5.0
LVM% (%)	83 ± 18	Mean 24-hour HR (bpm)	68 ± 10
Inadequate LVM (n)	7/25	24-hour SDNN (msec)	177 ± 36
E/A	2.1 ± 0.8	24-hour SDANN (msec)	161 ± 33
LVEF (%)	63 ± 9	VLF (ms^2^/Hz)	36 ± 8
Albumin (g/dL)	4.4 ± 0.6	LF (ms^2^/Hz)	31 ± 11
Cholesterol (mg/dL)	158 ± 30	HF (ms^2^/Hz)	22 ± 9

Data shown as Mean ± SD. SBP: systolic blood pressure; DBP: diastolic blood pressure; HR: heart rate; RWT: relative wall thickness; E/A: ratio of the E wave to A wave velocities of transmitral flow; LVEF: left ventricular ejection fraction; SDANN: SD of the averages of NN intervals in all 5-minute segments; SDNN: SD of all NN intervals; VLF: very-low frequency power; LF: low frequency power; HF: high frequency power.

**Table 2:** Comparison between patients with adequate and inadequate left ventricular mass.

	Adequate LVM (*n* = 18)	Inadequate LVM (*n* = 7)	*P* =
Age (years)	23 ± 6	26 ± 14	0.4
HR (bpm)	60 ± 11	54 ± 10	0.2
SBP (mm Hg)	93 ± 8	90 ± 7	0.4
DBP (mm Hg)	62 ± 8	64 ± 5	0.7
BMI	15.3 ± 1.6	13.9 ± 2.0	0.08
Albumin (g/dL)	4.5 ± 0.5	4.1 ± 0.8	0.2
Hemoglobin (g/dL)	12.3 ± 2	11.7 ± 3	0.4
GH (ng/mL)	7.1 ± 6.5	23.9 ± 25	0.03*
IGF-1 (ng/mL)	136.4 ± 50.9	103.6 ± 57	0.2
logIGF1/GH	1.4 ± 0.5	0.8 ± 0.7	0.02*
FT3 (pg/mL)	2.1 ± 0.3	1.3 ± 0.3	<0.001*
FT4 (ng/dL)	0.9 ± 0.1	0.9 ± 0.2	0.7
TSH (*μ*U/mL)	2.1 ± 0.5	1.9 ± 0.4	0.6
Cortisol (ng/mL)	161 ± 32	189 ± 44	0.1
FMD (%)	14.0 ± 5.8	13.5 ± 2.5	0.8
Mean 24-hour HR (bpm)	71 ± 8	64 ± 6	0.07
Age at onset (years)	15.3 ± 2.0	15.2 ± 1.6	0.8
Disease duration (years)	7.7 ± 5.6	11.0 ± 12.8	0.4

*Note.* See Table 1. *P < 0.05 at unpaired *t*-test.
